# Novel triadius-like N_4_ specie of iron nitride compounds under high pressure

**DOI:** 10.1038/s41598-018-29038-w

**Published:** 2018-07-13

**Authors:** Yuanzheng Chen, Xinyong Cai, Hongyan Wang, Hongbo Wang, Hui Wang

**Affiliations:** 1 School of Physical Science and Technology, Key Laboratory of Advanced Technologies of Materials, Ministry of Education of China, Southwest Jiaotong University, Chengdu, 610031 China; 2 State Key Lab of Superhard Materials, Jilin University, Changchun, 130012 China

## Abstract

Various nitrogen species in nitrides are fascinating since they often appear with these nitride as superconductors, hard materials, and high-energy density. As a typical complex, though iron nitride has been intensively studied, nitrogen species in the iron–nitrogen (Fe-N) compounds only have been confined to single atom (N) or molecule nitrogen (N_2_). Using a structure search method based on the CALYPSO methodology, unexpectedly, we here revealed two new stable high pressure (HP) states at 1:2 and 1:4 compositions with striking nitrogen species. The results show that the proposed FeN_2_ stabilizes by a break up of molecule N_2_ into a novel planar N_4_ unit (*P*6_3_/*mcm*, >228 GPa) while FeN_4_ stabilizes by a infinite 1D linear nitrogen chains N∞ (*P*-1, >50 G*P*a; *Cmmm*, >250 GPa). In the intriguing N_4_ specie of *P*6_3_/*mcm*-FeN_2_, we find that it possesses three equal N = N covalent bonds and forms a perfect triadius-like configuration being never reported before. This uniqueness gives rise to a set of remarkable properties for the crystal phase: it is identified to have a good mechanical property and a potential for phonon-mediated superconductivity with a T_c_ of 4–8 K. This discovery puts the Fe-N system into a new class of desirable materials combining advanced mechanical properties and superconductivity.

## Introduction

Nitrogen (N) is the most abundant element in the earth’s atmosphere and is one of the least studied elements regarding the composition of the Earth^1^. At standard temperature and pressure (T = 298 K; P = 1 atm), elemental nitrogen is a gas, consisting of diatomic N_2_ molecules that are bound by stiff covalent triple bonds. So the molecule is chemically inert and hardly dissociate and not many higher molecular or extended structures are known for nitrogen other than N_2_ under normal conditions. Syntheses of useful nitrides with various nitrogen species rely on chemical methods via, e.g., photochemical reaction, electrochemical synthesis^2–8^. A few higher molecular units are known, such as photolytic cyclic N_3_^3,4^, the tetrahedral N_4_ molecule^5^, the N_5_^−^ anion^6^, and the N_5_^+^ in a crystalline phase of N_5_^+^SbF_6_^−^^7,8^. Note that, among these units, though the tetrahedral N_4_ has been a form of the N_4_ unit for synthesis, it is observed as a metastable species with a lifetime exceeding one microsecond.

Besides the chemical methods, in fact, adopting HP technology nitrogen also does form innumerable stable and metastable chemical compounds with various nitrogen species^9–17^. These nitrogen species have various structural forms ranging from single atom (N) to molecular (N_2_, N_3_, N_4_, N_5_, N_6_) units and polynitrides^13–17^. To note, these stable nitrides have a variety of intriguing properties, such as superconductivity (MoN)^13^, high-energy density (LiN_3_, NaN_3_)^14^, and high hardness (WN_2_)^15^, as well as extraordinary chemical and thermal stability (Xe-N)^16^. These nitrides aroused our significant interest in the field of exploring new HP nitrogen species and their potential remarkable properties.

As a typical transition-metal nitride, the Fe-N system is extensively investigated to explore its compounds in the interior layers of earth following its first discovery since 19 century^2^. A rich Fe-N chemistry exists, most synthesized compounds have a Fe/N ratio higher than unity, such as *α*″-Fe_16_N_2_, *α*′-Fe_8_N, *γ*′-Fe_4_N, Fe_7_N_3_, Fe_3_N_x_(x = 0.75–1.4), Fe_3_N, Fe_2_N and FeN^4–10^. Among, the FeN compound is most nitrogen-rich iron nitride reported benign synthesized in a high pressure apparatus thus far^10^. This synthesis of FeN spurred the endeavors in search for Fe-N compounds with a more nitrogen content exceeding the FeN compound with other nitrogen species. However, in contrast to the Fe-rich compounds, there is little work on the N-rich iron nitrides, both from the experimental and theoretical sides. Only few theoretical investigations are available to report that an N-rich iron pernitride (FeN_2_) crystallizes in the space group *R*-3*m* at 17 GPa (1000 K)^11^ and transforms an orthorhombic *Pnnm* structure up to 22 GPa^12^ obtained by assuming the parent metal under pressure. All these known Fe–N compounds adapt single N atom or molecule N_2_ configuration and keep iron 6-coordination.

In order to systematically explore the possibility of obtaining new stable N-rich iron nitrides, and especially to examine the possibility of attaining new nitrogen species at HP, we here present extensive structure searches of stoichiometric Fe-N compounds under various pressures ranging from 0 to 300 GPa, using an unbiased particle swarm optimization (PSO) algorithms for crystal structure predictions^18^. This swarm-intelligence high-throughput searching has proven effective in revealing new compositions favorable to form in large sets of multicomponent Ca-H, Li-B, Xe-N, Cs-N systems^16,17,19,20^. The effectiveness has been also demonstrated by recent successes in predicting high-pressure structures of various systems, and their several experimental confirmations^21–30^. In this work, we proposed new N-rich iron nitrides at 1:4 and 1:2 compositions under HP. Identifying their nitrogen species, it is strikingly found that the nitrogen species evolve from a N_2_ unit to a novel N_4_ units, and eventually N∞ with the increase of N contents. In N_4_ unit, we find that it possesses three N = N covalent bonds and one lone pair, which leads it to forms an unknown triadius-like configuration. Its structural uniqueness gives rise to a set of remarkable properties for the crystal *P*6_3_/*mcm* phase with an unexpectedly Tc of 4~8 K and a good mechanical property.

## Methods

The developed CALYPSO structure prediction method designed to search for the stable structures of given compounds has been employed for the investigation of phase stability of Fe-N systems in N-rich stoichiometry under HP. We performed structure predictions of stoichiometric Fe_1-i_N_i_ (0 < i < 1) with simulation cell sizes of 1–4 formula units (f.u.) in a pressure range from 0 to 300 GPa. The local structural relaxations and electronic band structure calculations were performed in the framework of density functional theory within the generalized gradient approximation (GGA) and frozen-core all-electron projector-augmented wave (PAW) method^31,32^, as implemented in the VASP code^33^. The PAW pseudopotentials with 3*d*^7^4*s*^1^ and 2*s*^2^2*p*^3^ valence electrons were adopted for Fe and N, respectively. The kinetic energy cutoff for the plane-wave basis set is taken as 800 eV and a dense k-point grid with the spacing of 2π × 0.03 Å^−1^ was used to sample the Brillouin zone, which was shown to yield excellent convergence for total energies (within 1 meV/atom). The phonon calculations were carried out by using a finite displacement approach through the PHONOPY code^34^. The electron-phonon coupling (EPC) of *P*6_3_/*mcm*-FeN_2_ was calculated within the framework of linear response theory through the Quantum-ESPRESSO code^35^. A 2 × 2 × 2 q mesh was used in the interpolation of the force constants for the phonon dispersion curve calculations. A MP grid of 12 × 12 × 12 was used to ensure k-point sampling convergence, which approximates the zero-width limits in the calculations of EPC parameter. We Elastic constants were calculated by the strain-stress method and the bulk modulus and shear modulus were thus derived from the Voigt-Reuss-Hill averaging scheme^36^.

## Results and Discussions

**Figure 1:**
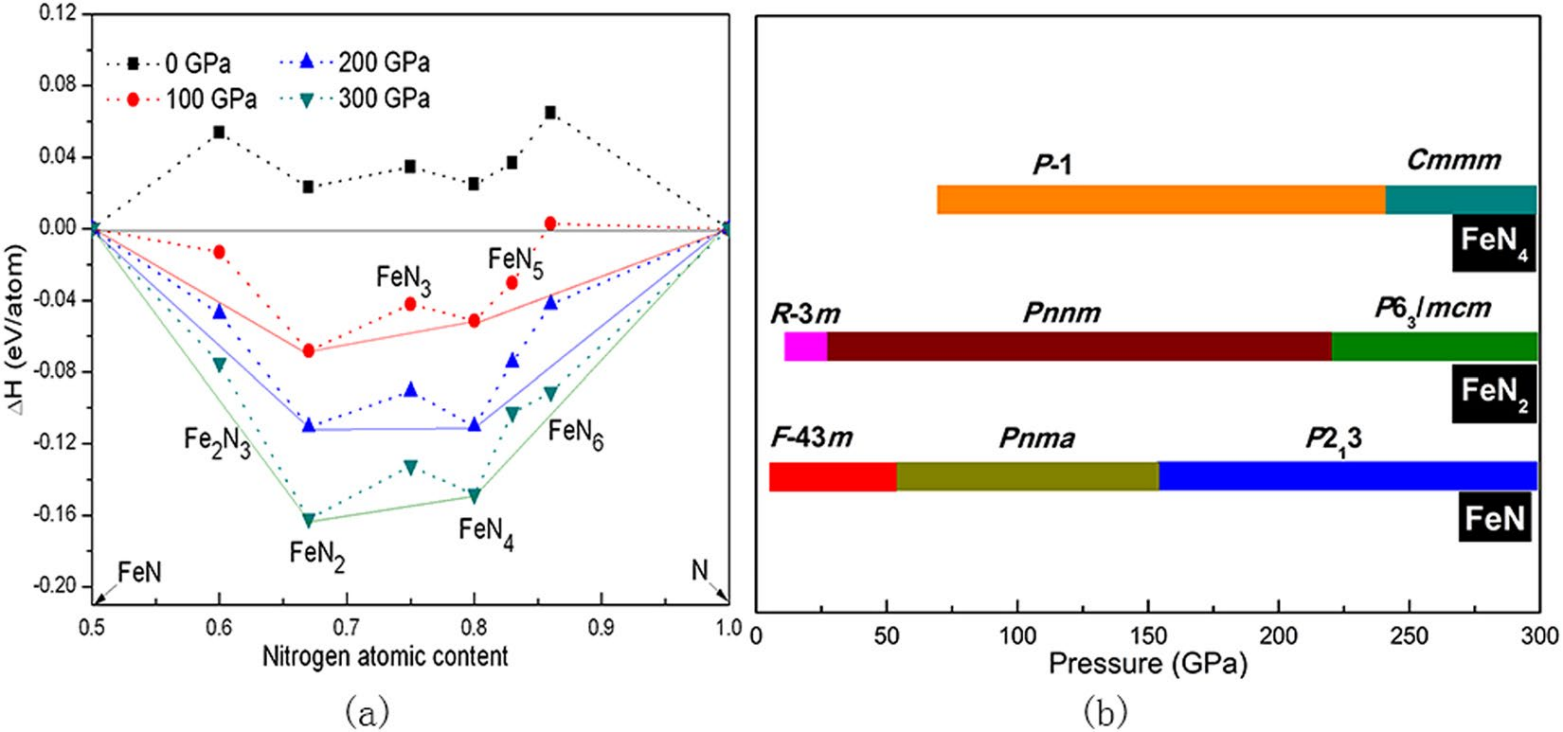
(**a**) Formation enthalpies (ΔH) of various Fe-N compounds with respect to decomposition into constituent elemental solids at 0–300 GPa. Data points located on the convex hull (solid lines) represent stable species against any type of decomposition. (**b**) Pressure ranges in which the corresponding structures of FeN, FeN_2_ and FeN_4_ are stabilized.

We focused our structure search on the phase stabilities of Fe-N systems in N-rich stoichiometry by calculating the formation enthalpy of various Fe_1-i_ N_*i*_ (0 < *i* < 1) compounds in a pressure range of 0 to 300 GPa. The formation enthalpy was calculated with respect to the decomposition into FeN and N, as Δ*h*(Fe_1-i_ N_*i*_) = *h*(Fe_1−i_ N_*i*_) − (1−*i*)*h*(FeN) − (*2i*−1)*h*(N), where the enthalpies *h* for Fe_1-i_ N_*i*_ and FeN are obtained for the most stable structures as searched by the CALYPSO method at the desired pressures. The convex hulls are depicted in Fig. 1a for pressures at 0, 100, 200 and 300 GPa. The validity of using FeN instead of Fe in defining Δ*h* is ensured by the fact that FeN is exceedingly stable with respect to the binary Fe-N system having a max nitrogen ratio below 50% (Fe_4_N, Fe_3_N, Fe_2_N) under HP, as revealed by our study (Fig. S1) and relative experimental studies^4–7,10^. The stable structure of FeN below 50 GPa has space group *F*-43*m* structure (Fig. S2, ref.^10^). At 50 GPa, the FeN transforms into a *Pnma* structure (Fig. S3), followed by a cubic *P*2_1_3 structure above 150 GPa (Fig. S4). All these FeN structures takes on isolated N atomic sublattice and keeps six-fold coordinated by Fe forming edge-sharing FeN_6_ octahedron in their corresponding stable pressure ranges.

Magnetism plays a central role in iron and its compounds. Therefore it is necessary to confirm the role of magnetism on the stability of these Fe-N structures. From our spin-polarized calculations, we find that every Fe atom of Fe-N compounds possesses a magnetic moment of 0.21–1.68 μ_B_ under pressure (<50 GPa), which is substantially lower than that of the pure Fe solid (2.2 μ_B_). Meanwhile, the magnetic moment will decrease rapidly with increasing pressure and be completely quenched as pressure exceeds 50 GPa. As an example, we performed the energy calculations of FeN after considering magnetism and found that the magnetic effect did not change the phase transition sequence but slightly shifted the phase transition pressure. According to a model derived from a Slater-Pauling type behavior^37^, the magnetization with increasing amount of N becomes decrease in the Fe–N system. It thus is plausible to perform the structure search and enthalpy calculations without considering the magnetic effect under HP in the N-rich Fe–N compounds.

Analysis the convex hull for researching the thermodynamically stable in the Fig. 1a, we can get a main result as follows: at *P* = 0 GPa, the Δ*h* of all N-rich stoichiometry are positive, meaning that the nitrogen ratio above 50% Fe-N system are not stable. This is consistent with the experimental observation that no Fe-N compound whose which the nitrogen content exceeds the iron content can form at ambient pressure; at 100 GPa, stable stoichiometries of FeN_2_ and FeN_4_ emerge on the convex hull as the most stable stoichiometry, this situation preserves up to 300 GPa. Detailed pressure-composition phase diagram for these two N-rich species is presented in Fig. 1b. Moreover, we performed phonon spectra calculations using the finite-displacement method to assess the dynamical structural stability of their structural phases at desired pressure. No imaginary frequency was found for their structures, which indicated that they are dynamically stable (Figs S5–10).

**Figure 2:**
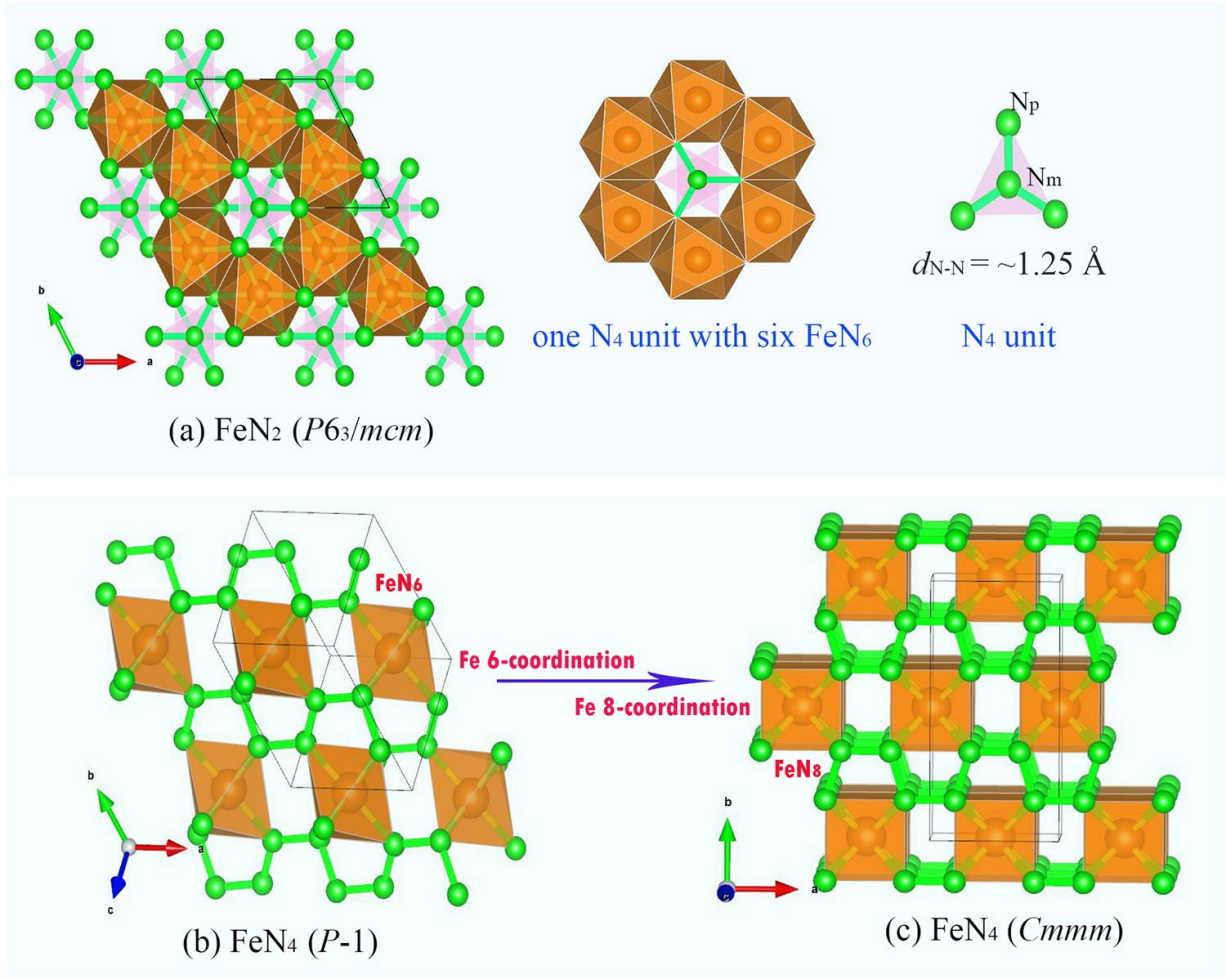
Structures of predicted stable FeN_2_ and FeN_4_ crystals: (**a**) The *P*6_3_/*mcm* structure of FeN_2_, viewing of the FeN_6_ and N_4_ unit. (**b**) Low pressure *P*-1 structure of FeN_4_. (**b**) the HP *Cmmm-*FeN_4_.

In the FeN_2_ compound, the low pressure crystalline phase is a trigonal *R*-3*m* structure at approximately 22 GPa, above which an orthorhombic *Pnnm* structure becomes more favorable, consistent with the previous reports^11,12^. These two structures both contain a dinitrogen unit and N-sharing six-fold FeN_6_ octahedrons (Figs S11,12). Analysis of the dinitrogen unit indicating a strong N-N covalent bond and the dinitrogen unit can be formulated as (N-N)^2−^ and (N = N)^2−^ in the *R*-3*m* and *Pnnm* structures, respectively^11,12^. Recently, these phases have been synthesized and verified by the experiment under high pressure and high temperature^38^. Upon to 228 GPa, an unknown energetically more favored hexagonal *P*6_3_/*mcm* structure was firstly discovered (Fig. 2a). Tracing the volume change of the phase transition from *Pnnm* to *P*6_3_/*mcm*, it is found that this transition is a first-order accompanied by a volume drop of 3.5%. Viewing the *P*6_3_/mcm structure, it contains two types of N atoms occupying two different 2*c* Wyckoff sites as middle N_m_ and peripheral N_p_ (Fig. 2a): the N_p_ atom is shared by four Fe atoms forming a FeN_6_ octahedron with Fe-N_p_ distances of 1.76 Å, while the N_m_ atom bonds with three N_p_ atoms forming perfect N_4_ unit with a bond length of ~1.25 Å (at 300 GPa). These Fe-N_p_ distances are shorter than the sum (1.92 Å) of covalent radii of Fe and N atoms. In such exotic structure, each Fe forms 6 Fe-N_p_ bonds with those 6 neighboring N_p_ atoms and each N_p_ atom has four neighbors Fe-N_p_ bonds and a N_p_-N_m_ bond. The N_p_-N_m_ distance (1.25 Å) is slightly longer than the double N = N bonds (1.20 Å) can be verified as the N = N bonding nature.

In the FeN_4_ compound, the energetically favored structure of FeN_4_ (stable above 50 GPa) has a triclinic structure with a low *P*-1 symmetry (Fig. 2b). The nitrogen sublattice in this structure takes on a polyacetylene-like infinite linear chain structure with a closest NN separation (N∞) in the range of 1.32−1.34 Å. Polyhedral view of the *P*-1 structure, it forms octahedrons linked together by the NN bonds of N∞, with Fe atoms sitting at the center of octahedrons and being 6-fold bonded to the N atoms of N∞. Up to 250 GPa, a surprising transition from such the 6-fold *P*-1 structure to a 8-fold structure takes place. This 8-fold structure adopts a high symmetric orthorhombic *Cmmm* structure, which have similar N∞ structural character of the *P*-1 phase (Fig. 2c). Analysis of their ELF (Figs S15,16) suggests that the N atoms of N∞ are in *sp*^2^ hybridization, each N forms two σ bonds with two neighboring N atoms and one Fe-N bond. Due to the N∞ units, the electronic structures of these two phases both exhibit the metal properties (Figs S17,18). Such N∞ units and electronic properties can be also found in works for LiN_3_, NaN_3_, and CsN_3_ under high pressure^39–41^. For the two phases, their different is that the *Cmmm* phase are composed of a Fe 8-coordination decahedrons, in stark contrast with 6-coordination in the octahedrons of *P*-1 structure (Fig. 2b,c). Analyses of the coordination number of Fe, we note that conventional coordination chemistry of Fe consists of four-, five-, and six-coordinate metal ions, while coordination numbers higher than six are seldom observed in only discrete molecules and polynuclear metal clusters^42–49^. Despite much effort, the Fe atoms are found to be very resistive to become 8-fold coordinate in solids, and the search for solids containing 8-coordinate has so far been scarcely successful. To our knowledge, this is first time to identify the 8-fold coordination of Fe atoms in the Fe-N compounds.

**Figure 3:**
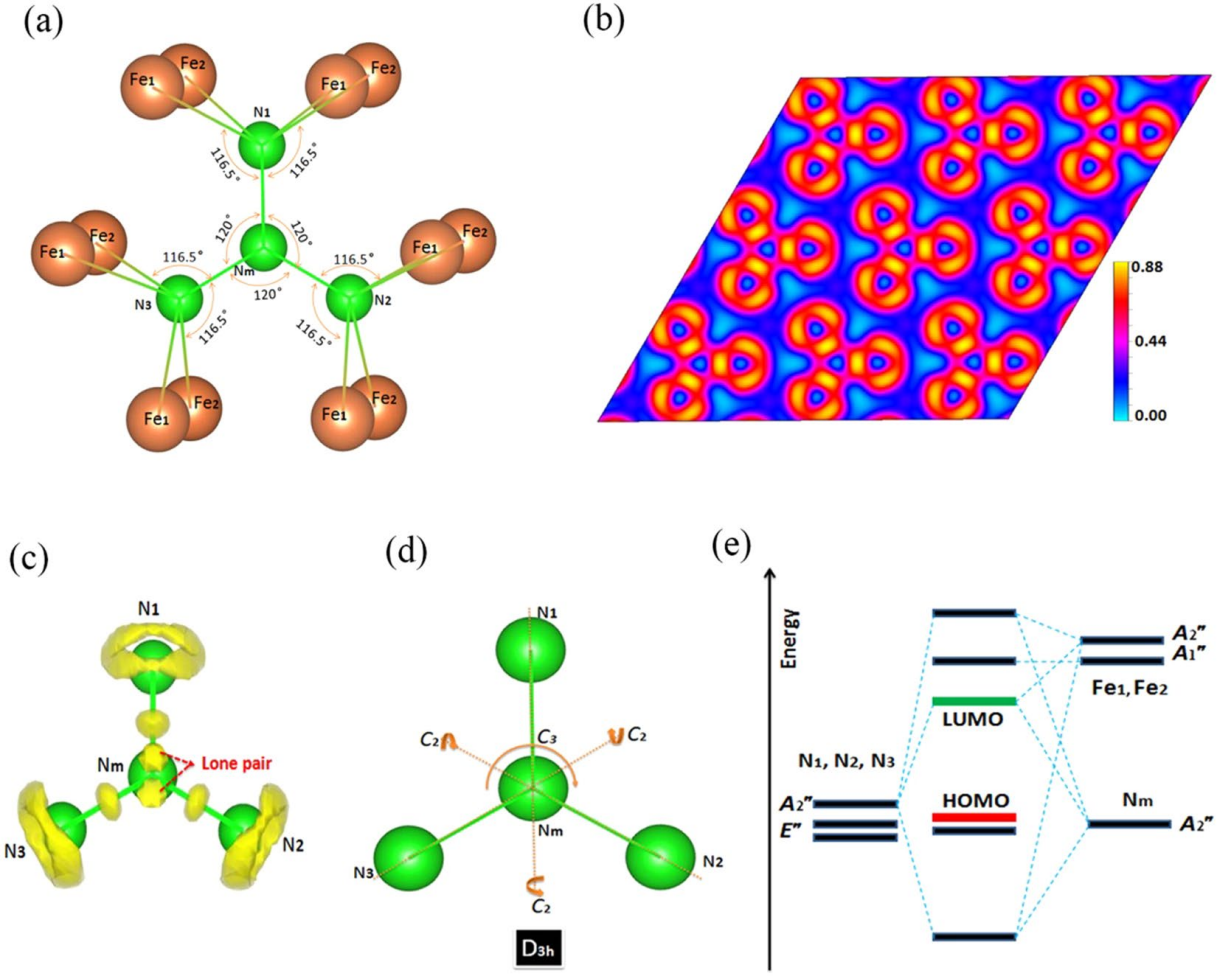
(**a**) The structural feature of N_4_ unit with Fe in the *P*6_3_/*mcm*-FeN_2_. (**b**) The ELF plots (001) of *P*6_3_/*mcm* structure at 250 GPa with an isosurface value of 0.75. (**c**) The ELF plots of N_4_ unit in the *P*6_3_/*mcm* structure at 250 GPa. (**d**) The atomic model for hypothetic N_4_ cluster with typical symmetry operations marked out. (**e**) The orbital interaction diagram of a N_4_ unit with Fe atom.

Return to identify the nitrogen species of Fe-N compounds (FeN, FeN_2_ and FeN_4_), it is strikingly found that the nitrogen sublattice evolve from isolated N atom to in turn the N_2_ unit, the N_4_ unit and eventually N∞ with the increase of N contents. It is noted that these features, except for the N_4_ units, can be often found in alkali metal azides (Li-N, Na-N, Cs-N)^39–41^. Tracing the history of the related N_4_ units, the first investigation into N_4_ units can be traced back to a reported about successfully isolated neutral N_4_ molecule in the gas phase a decade ago^5^, but the lifetime of the N_4_ molecule is only around 1 microsecond. Recently, a charged N_4_ species as predicted in the CsN crystal is substantially stabilized by strong cation-anion interactions^17^. This predicted N_4_^4−^ anion has an open-chain structure containing two terminal single-bonds and one internal double-bond. Being different that, we found here the N_4_ units of *P*6_3_/*mcm*-FeN_2_ has three equal N = N bond and forms plane N_4_ units like as the triadius star (Fig. 3a). We also try to look for crystal structures that incorporate such special N_4_ units in the other systems. But no crystal structure is found so far. Here the exotic N_4_ unit having strong N-N covalent bonding can be clearly shown by its ELF (Fig. 3b,c). Each N_m_ atom possesses one lone pair of electrons at its *p*_*z*_ orbital and forms three N = N covalent bonds with peripheral N_p_ (N1, N2, N3) atoms (Fig. 3c), owing to its *sp*^2^ hybridization.

Notice that the *p*_*z*_ orbital of N is much lower in energy than that of Fe and form a strong overlapping between *p*_*z*_ orbitals of N. This fact justifies us to study the electronic structure of hypothetic neutral N_4_ unit first before researching the electronic properties of its crystal *P*6_3_/*mcm* structure. The atomic model for neutral N_4_ unit is sketched in Fig. 3d with several typical symmetry elements of point group D_3h_ marked out explicitly (Table S7). The linear combination of three *p*_*z*_ orbitals of peripheral N_p_ can form orbitals with A_2_″ and E″ symmetry, while the p_z_ orbital of N_m_ belongs to A_2_″ (Table S8). Therefore, for a bare N_4_ unit, the four p_z_ orbitals of N would constitute two nonbonding (E″), one bonding and one antibonding molecular orbitals. According to the diagram in Fig. 3e, the HOMO derives from the nonbonding p_z_ orbitals of N_p_ atoms, while the LUMO comes mainly from the antibonding of *p*_*z*_ orbitals between N_m_ and N_p_. Meanwhile, the bader charge analysis reveals the fact of electron abundant N_4_ units and electron deficient Fe atoms (the less electronegative Fe loses 1.67 electrons per atom, and N_p_ directly bonded to Fe obtains 1.13 electrons per atom, while the N_m_ atom remains almost neutral). Such unoccupied antibonding orbitals between N_m_ and N_p_ can minimize the influence of excess electrons, which can explain why the *P*6_3_/*mcm* structure with the N_4_ unit is fairly stable.

**Figure 4:**
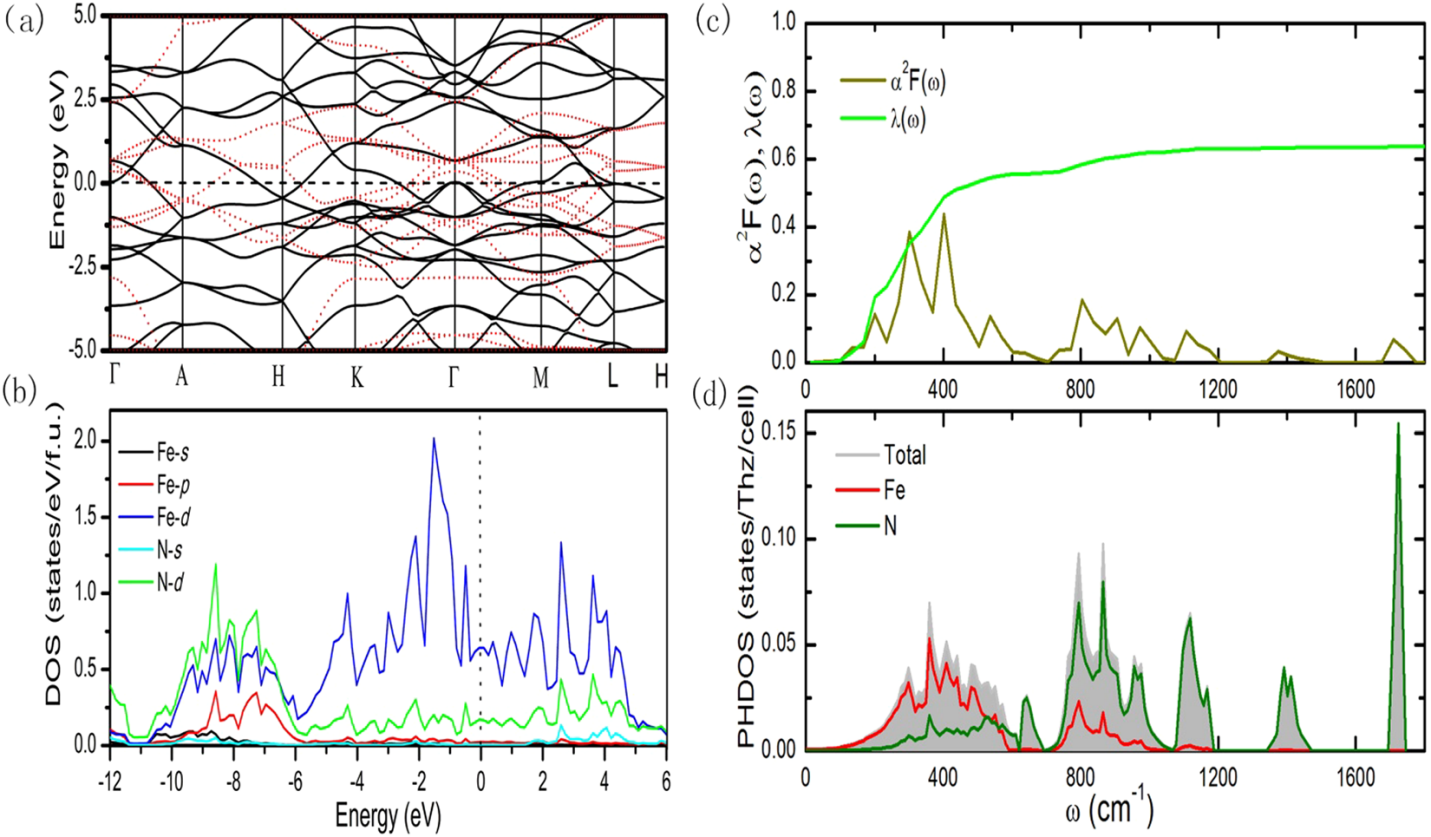
(**a**) Electronic band structure and (**b**) the projected density of states for *P*6_3_/*mcm*-FeN_2_ structure at 250 GPa, the red dash lines represent the band structure of the N sublattice with a uniform compensated background. (**c**) The calculated Eliashberg EPC spectral function α^2^F(ω) and its integral λ(ω) and (**d**) the projected phonon density of states for *P*6_3_/*mcm*-FeN_2_ structure at 250 GPa.

To probe the electronic structures of the *P*6_3_/*mcm* structure, we calculated the band structure and density of states (DOS), finding that it exhibit metallic features. A comparison of the band structures of FeN_2_, Fe_0_N_2_ (all Fe atoms removed out of the lattice and a uniform compensated background charge (8e/Fe) is applied to preserve the total valence electrons of the system) is performed (Fig. 4a). The difference between the resultant band structure of a hypothetical Fe_0_N_2_ system (red dash lines) with the realistic one of FeN_2_ indicates that the Fe atoms not only act as electron donors but also bond with N. As shown in Fig. 4b, the DOS reveal that the Fe-*d* and N-*p* states are energetically degenerate in the valence bands region, which facilitates the Fe−N hybridization and the formation of covalent bond. These results offer further support for the ionic and covalent bonding nature of Fe-N bonds as described above. We noted that the large bands crosses the *E*_f_ and the bands appears “flat band-steep band” characteristic^50^ around the *E*_f_. These are typical features favorable for strong EPC and superconductors. Using the linear response theory, we calculated the phonon DOS (PHDOS), Eliashberg function α^2^F(ω) and the strength of the e-ph coupling λ(ω) of the *P*6_3_/*mcm* structure (Fig. 4c). The Eliashberg function integrates to a λ = 0.62 and gives the logarithmic average ~400 cm^−1^, being much closer to the *oP*10-FeB_4_ value of ~430 cm^−1^. The main contributor to the EPC originates from the mixed Fe-N modes below 1000 cm^−1^ (85% of λ) and the high-frequency vibrations from N_4_ units (15% of λ) (Fig. 4d). Using the Allen-Dynes equation^51^, with the calculated ω_log_ of 643 K and typical µ* = 0.1~0.15, it reveals that the *P*6_3_/*mcm* structure is a weak-coupling BCS-type superconductor with a superconducting T_c_ of 4~8 K. Moreover, the most commonly known transition-metal pernitrides crystallize can act as hard materials, such as MnN_2_ (H_V_ = 19.9 GPa), CoN_2_ (16.5 GPa), and NiN_2_ (15.7 GPa)^52^. Fe in the same period as Mn, Co and Ni and the *P*6_3_/*mcm*-FeN_2_ phase is as a typical transition-metal pernitride, so the *P*6_3_/*mcm*-FeN_2_ is also regarded as hard material here. We calculated and obtained its mechanical properties including bulk modulus B (341 GPa), shear modulus G (247 GPa), young’s modulus Y_m_ (597 GPa), and Vicker’s hardness (H_V_ = 29 GPa) at ordinary pressure. As expect, the result shows that the *P*6_3_/*mcm*-FeN_2_ phase exhibits highly incompressible. Bases on a correlation the covalent bond with hardness, we attribute the excellent mechanical properties of this structure to the N_4_ units with strong covalent bonds dominantly by providing coulomb repulsion between the nitrogen atoms as a result of charge transfer from Fe.

## Conclusion

Using a structure search method based on CALYPSO methodology and density functional total energy calculations, we systematically studied the phase stabilities and the structures of Fe-N systems in the N-rich regime. We identify two stoichiometric FeN_4_ and FeN_2_ compounds with unexpected structures that might be experimentally synthesizable under pressure. At 1:4 composition, the energetically favored structure stabilizes in a low *P*-1 symmetry at low pressure and adopts a high symmetric orthorhombic *Cmmm* structure at high pressure, both having a infinite 1D linear nitrogen chains. Differently, the *Cmmm* phase has Fe 8-coordination decahedrons, in contrast with Fe 6-coordination in the octahedrons of *P*-1 structure. At 1:2 composition, an unknown energetically favored hexagonal *P*6_3_/*mcm* structure was firstly discovered at above 228 GPa. Structurally, it is intriguing with the appearance of exotic triadius-like N_4_ unit. In the N_4_ unit, the N_m_ atom possesses one lone pair of electrons at its *p*_*z*_ orbital and forms three N = N covalent bonds with peripheral N_p_ atoms, owing to its sp^2^ hybridization. To probe the electronic structures of the *P*6_3_/*mcm* structure, it reveals that its intriguing feature gives rise to a set of remarkable properties with an unexpectedly T_c_ of 4~8 K and a good mechanical property.

## Electronic supplementary material

Supplementary Information
