# Supplementary material for: Novel triadius-like N4 specie of iron nitride compounds under high pressure
Source: Sci Rep. 2018 Jul 13;8:10670. doi: 10.1038/s41598-018-29038-w (PMC6045616; doi:10.1038/s41598-018-29038-w)
Supplement: Supplementary file 1 — Supplementary Information [file 41598_2018_29038_MOESM1_ESM.doc]

**Supporting information**

Novel triadius star -like N_4_ specie of iron nitride compounds under high pressure

Yuanzheng Chen,^†^,* Xinyong Cai,^†^ Hongyan Wang,^†^ Hongbo Wang,^‡^,* and Hui Wang^‡^

^†^ School of Physical Science and Technology, Key Laboratory of Advanced Technologies of Materials, Ministry of Education of China, Southwest Jiaotong University, Chengdu 610031, China.

^‡^ State Key Lab of Superhard Materials, Jilin University, Changchun 130012, China

*Email of corresponding author: cyz@calypso.org.cn; webpage: http://mym.calypso.cn

**1. Computational details**

We employ a global minimization of free energy surfaces based on *ab initio* density functional (DFT) total-energy calculations and a particle swarm optimization (PSO) algorithm as implemented in the CALYPSO code. Structure searches were performed at 0, 100, 200 and 300 GPa with up to four formula units for Fe_1-i_N_i_ (0 < i < 1). In the first step, random structures with certain symmetry are constructed in which the atomic coordinates are generated by the crystallographic symmetry operations. Then the structures are optimized to local minima by using DFT (VASP code) calculations. After processing the first generation structures, 60% of them with lower enthalpies are selected to produce the next generation structures by PSO. 40% of the structures in the new generation are randomly generated. A structure fingerprinting technique of bond characterization matrix is applied to the generated structures, so that identical structures are strictly forbidden. These procedures significantly enhance the diversity of the structures, which is crucial for the efficiency of the global search of structures. The local optimizations are performed by use of the conjugate gradients method and the criteria of the enthalpy change is 2 × 10^-5^eV per atom. For most of the cases, the structure searches reach the convergence after 35 generations covering about 2000 structures.

**2. Computational data (Table and Figure)**

**Table S1**. Detailed structure information of *Pnma* for FeN at 100 GPa.

| a, b, c [Å] | 4.58700 | 2.52520 | 4.48190 |
| --- | --- | --- | --- |
| α,β,γ [°] | 90.0000 | 90.0000 | 90.0000 |
| Fe(4c) | 0.01088 | 0.25000 | 0.22102 |
| N(4c) | -0.28349 | 0.25000 | 0.92378 |

**Table S2**. Detailed structure information of *P*2_1_3 phase for FeN at 300 GPa.

| *a*, *b*, *c* [Å] | 3.64480 | 3.64480 | 3.64480 |
| --- | --- | --- | --- |
| α,β,γ [°] | 90.0000 | 90.0000 | 90.0000 |
| Fe(4a) | 0.95612 | 0.04388 | 0.54388 |
| N(4a) | 0.54204 | 0.45796 | 0.95796 |

**Table S3**. Detailed structure information of *P*nn*m* phase for FeN_2_ at 100 GPa.

| *a*, *b*, *c* [Å] | 3.75540 | 4.45700 | 2.41760 |
| --- | --- | --- | --- |
| α,β,γ [°] | 90.0000 | 90.0000 | 90.0000 |
| Fe(2d) | 0.00000 | 0.50000 | 0.50000 |
| N(4g) | -0.86913 | 0.09825 | 0.50000 |

**Table S4**. Detailed structure information of *P*6_3_/*mcm* phase for FeN_2_ at 300 GPa.

| *a*, *b*, *c* [Å] | 4.07060 | 4.07060 | 4.18760 |
| --- | --- | --- | --- |
| α,β,γ [°] | 90.0000 | 90.0000 | 120.0000 |
| Fe(4d) | 0.66667 | 0.33333 | 0.50000 |
| N1(2a) | 0.00000 | 0.00000 | 0.25000 |
| N2(6g) | 0.00000 | 0.30687 | 0.25000 |

**Table S5**. Detailed structure information of *P*-1 structure for FeN_4_ at 100 GPa.

| *a*, *b*, *c* [Å] | 3.58160 | 4.31010 | 4.46740 |
| --- | --- | --- | --- |
| α,β,γ [°] | 71.9162 | 90.2445 | 114.4450 |
| Fe( 2i) | 0.72952 | 0.73286 | 0.75828 |
| N1(2i) | 0.58771 | 0.08211 | 0.75396 |
| N2(2i) | 0.77725 | 0.63374 | 0.18895 |
| N3(2i) | 0.75681 | 0.88946 | 0.31712 |
| N4(2i) | 0.11948 | 0.60517 | 0.25575 |

**Table S6**. Detailed structure information of *Cmmm* phase for FeN_4_ at 300 GPa.

| *a*, *b*, *c* [Å] | 3.23330 | 6.52830 | 2.13160 |
| --- | --- | --- | --- |
| α,β,γ [°] | 90.0000 | 90.0000 | 90.0000 |
| Fe(2c) | 0.50000 | 0.00000 | 0.50000 |
| N(8p) | 0.19162 | 0.15647 | 0.00000 |


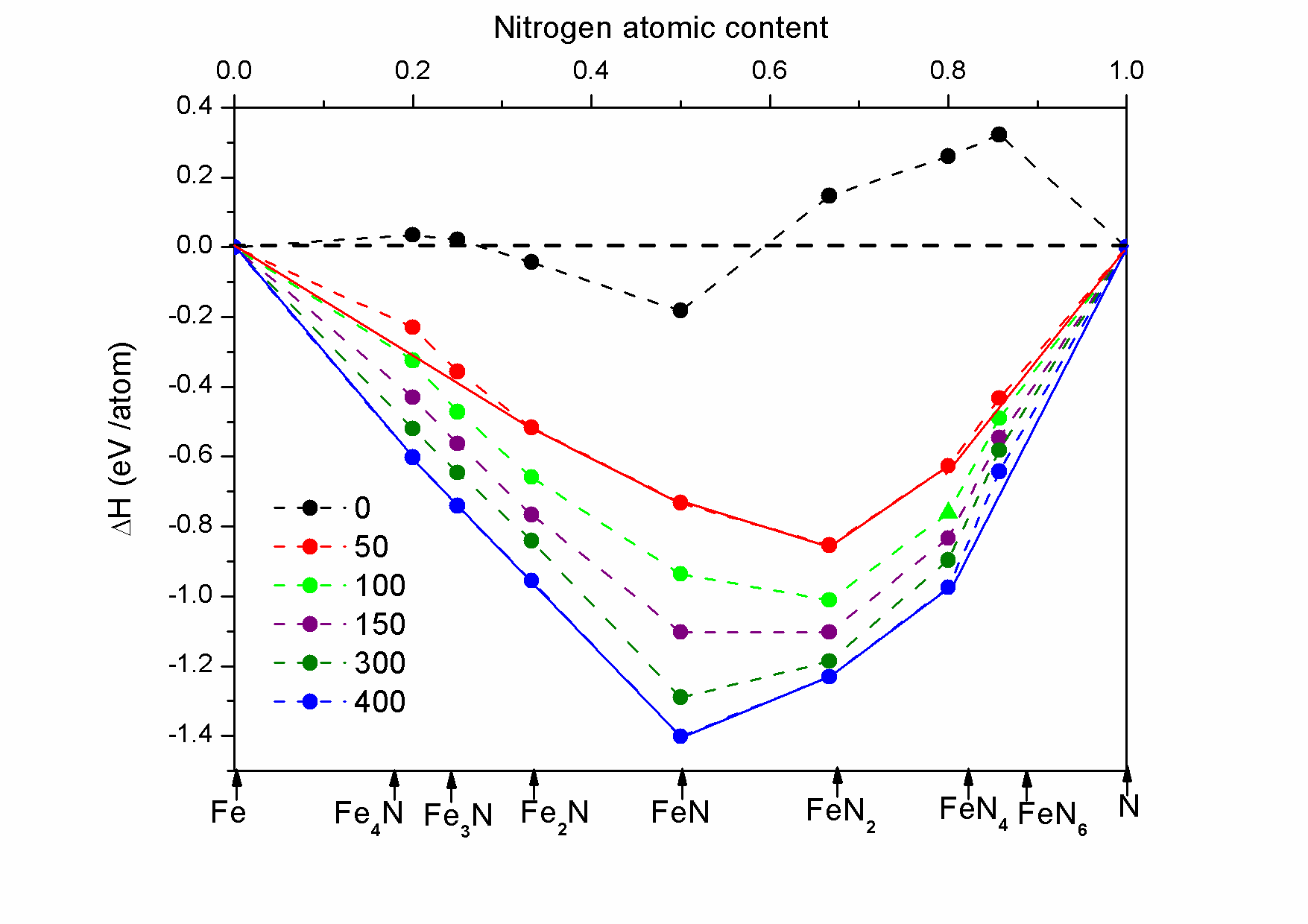


**Figure S1**. Formation enthalpies (ΔH) of various Fe-N compounds with respect to decomposition into constituent elemental solids at 0-400 GPa.


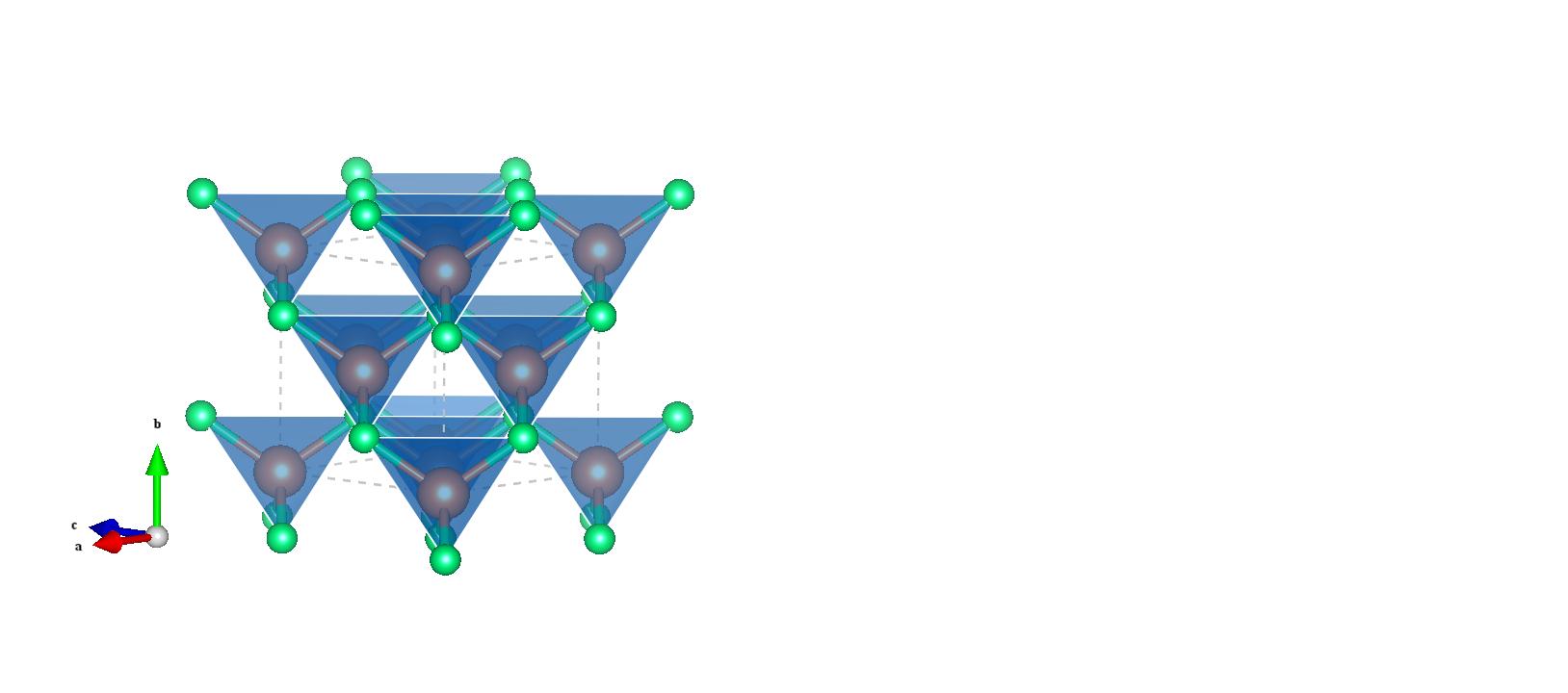


**Figure S2.** The *F-*43*m* structure of the stable FeN crystals


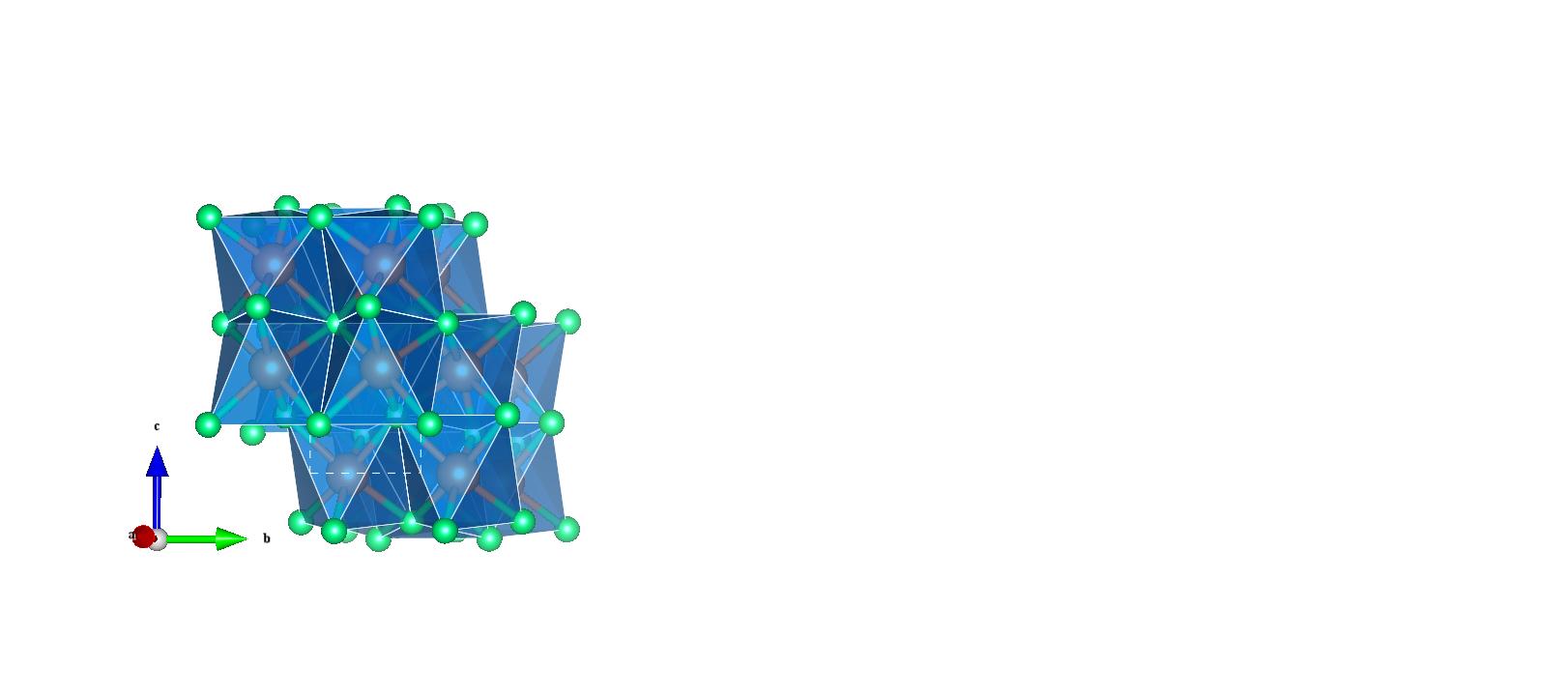


**Figure S3.** The *Pnma* structure of predicted stable FeN crystals

**
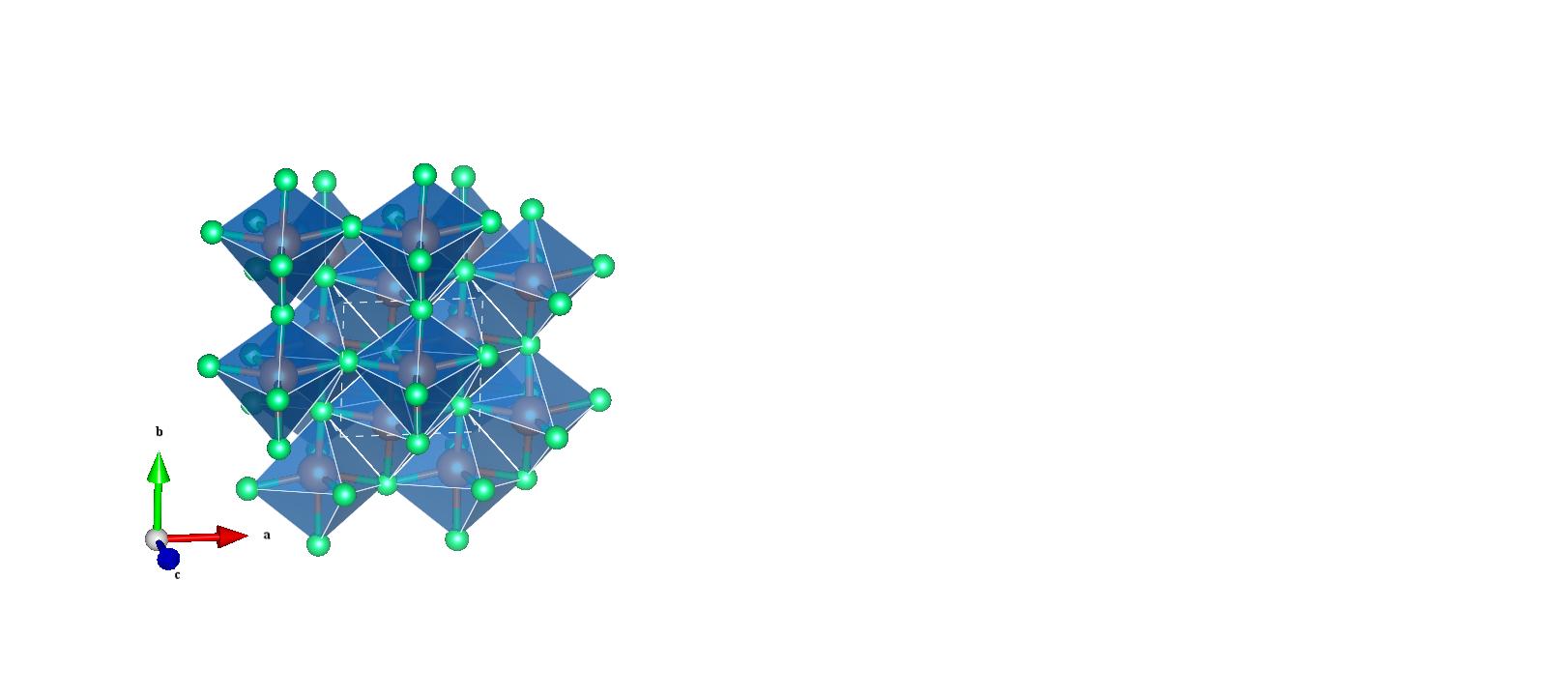
**

**Figure S4.** The *P*2_1_3 structure of predicted stable FeN crystals


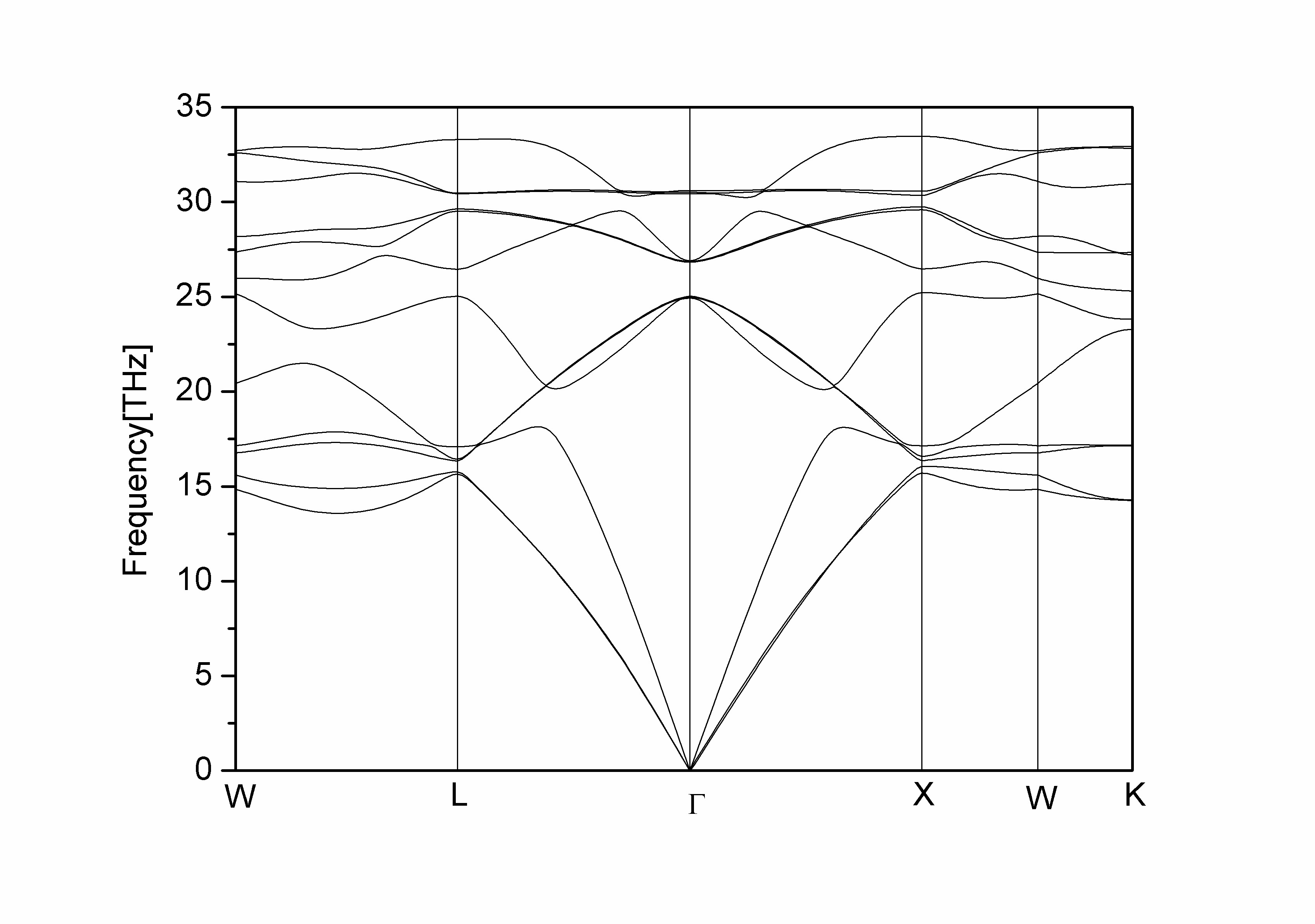


**Figure S5.** Phonon dispersion curves of *Pnma* structure for FeN at 50 GPa.


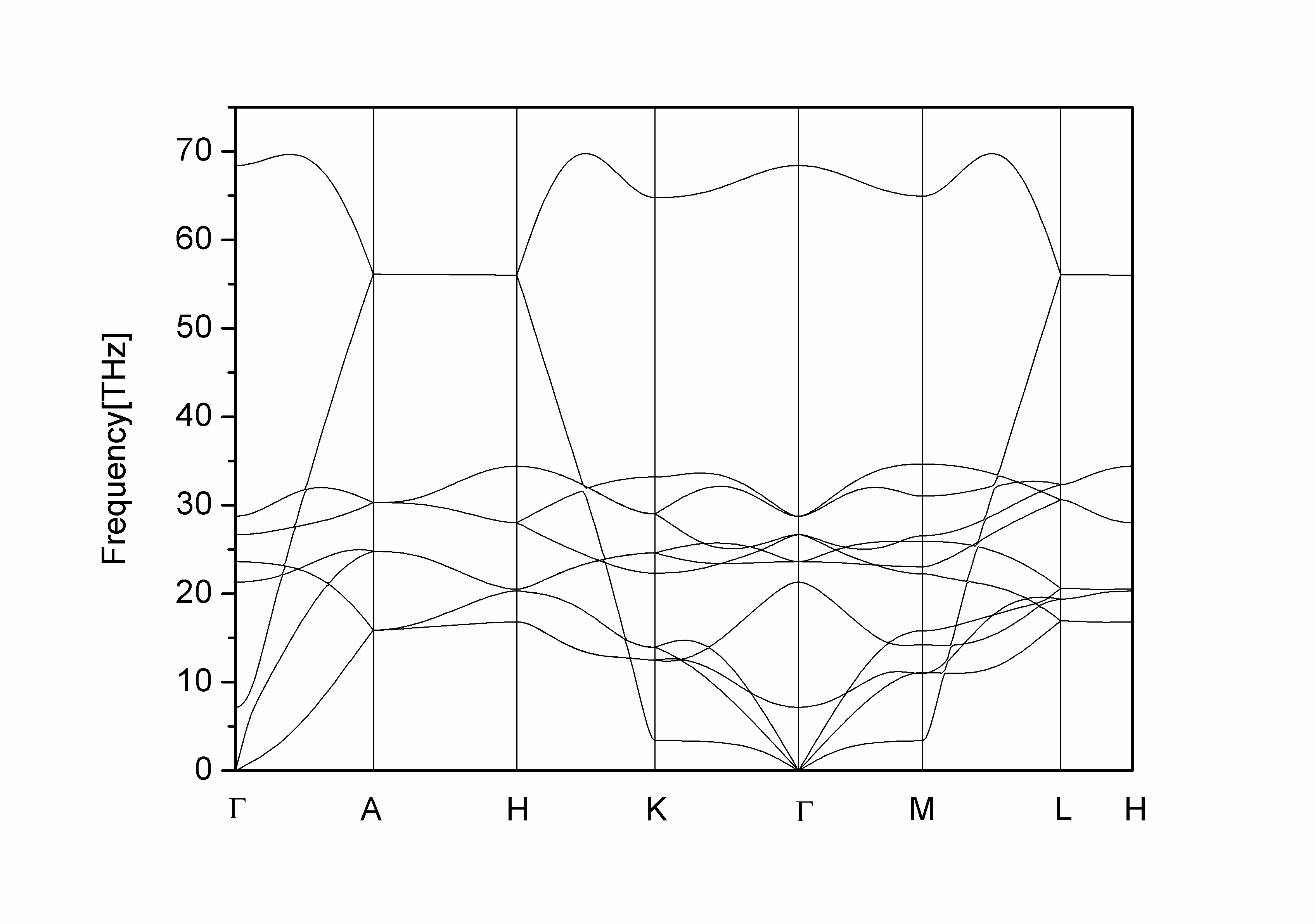


**Figure S6.** Phonon dispersion curves of *P*2_1_3 structure for FeN at 200 GPa.


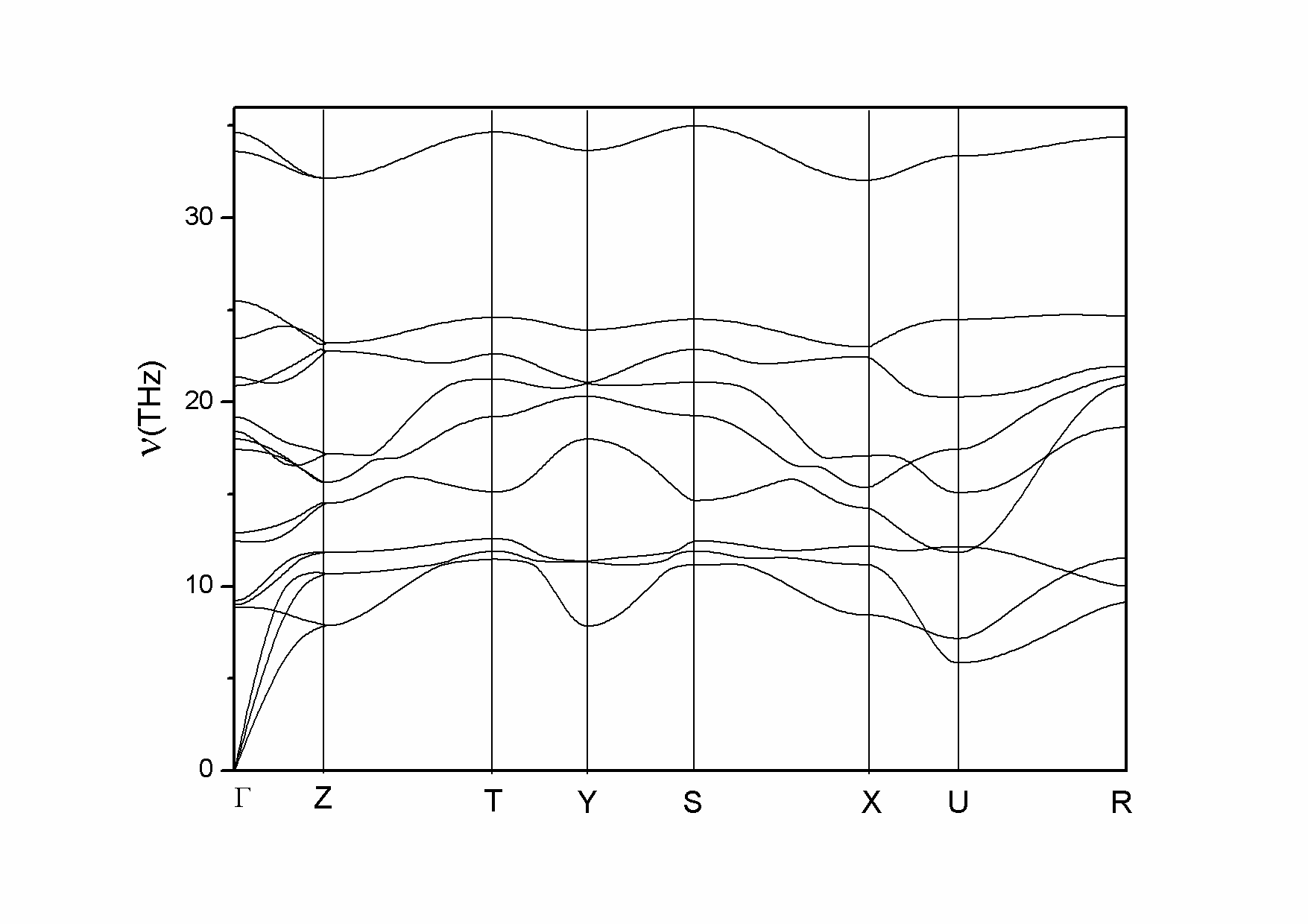


**Figure S7**. Phonon dispersion curves of *Pnnm* phase for FeN_2_ at 50 GPa.


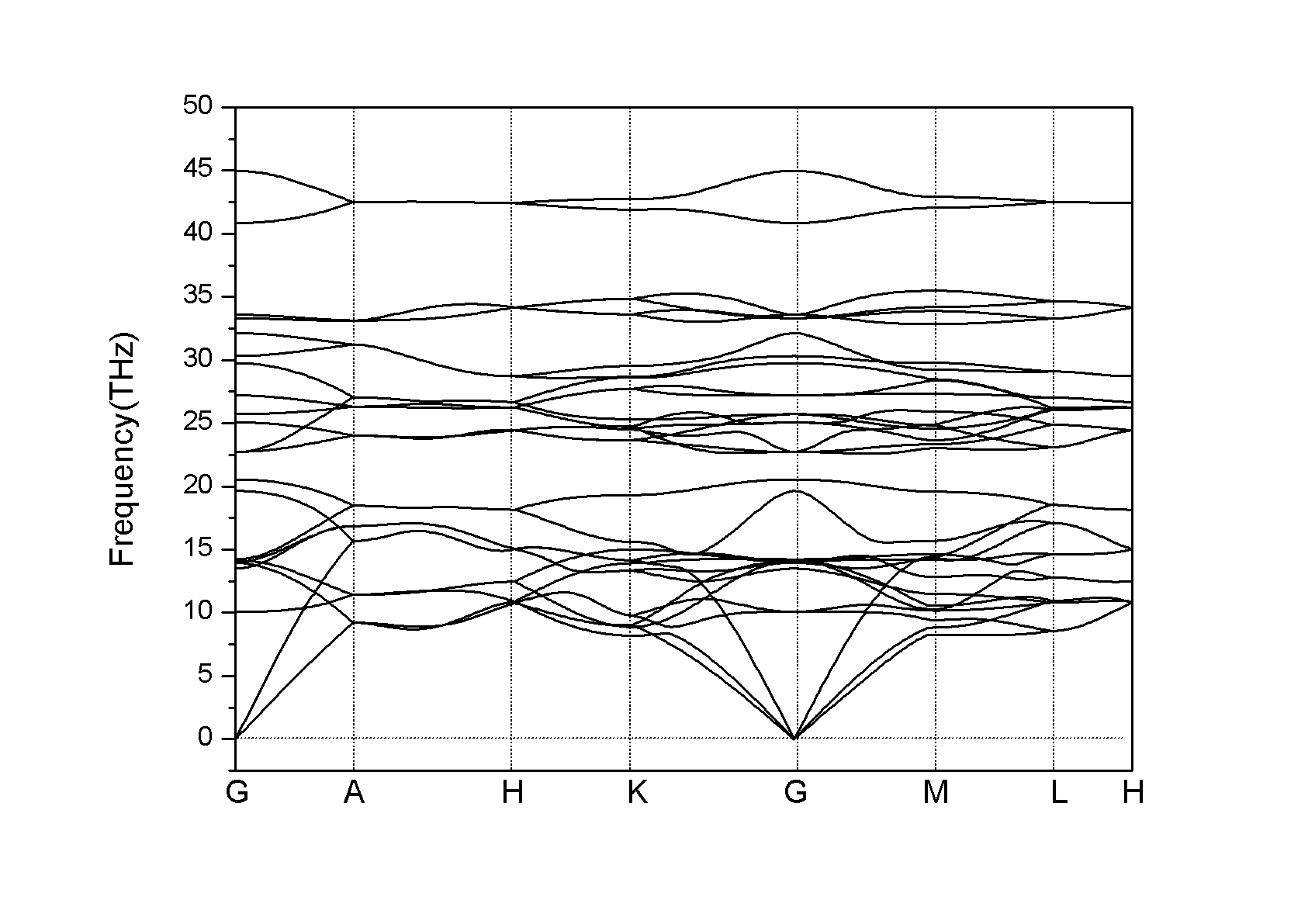


**Figure S8**. Phonon dispersion curves of *P*6_3_/*mcm* phase for FeN_2_ at 300 GPa.


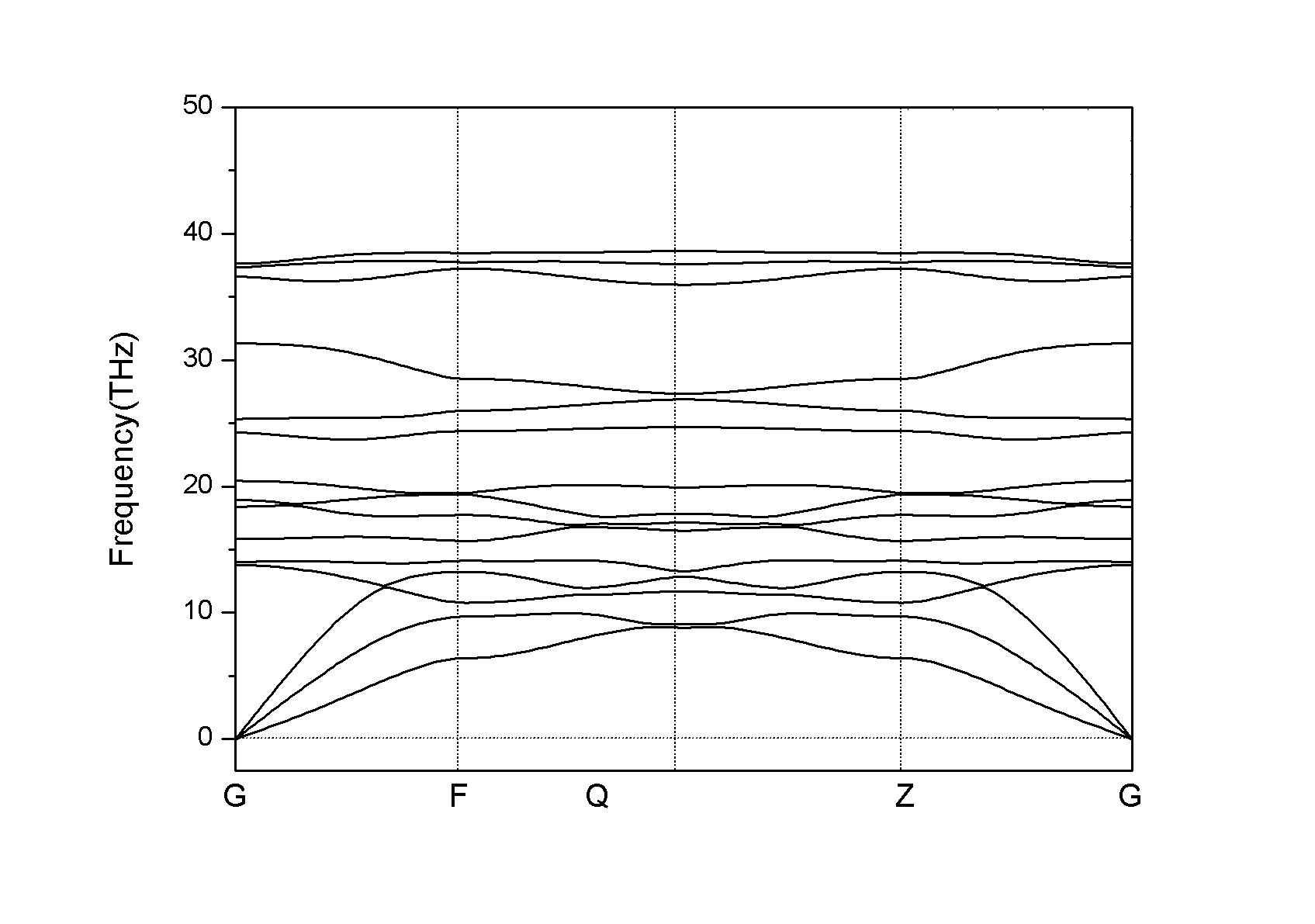


**Figure S9**. Phonon dispersion curves of *P*-1 phase for FeN_4_ at 150 GPa.


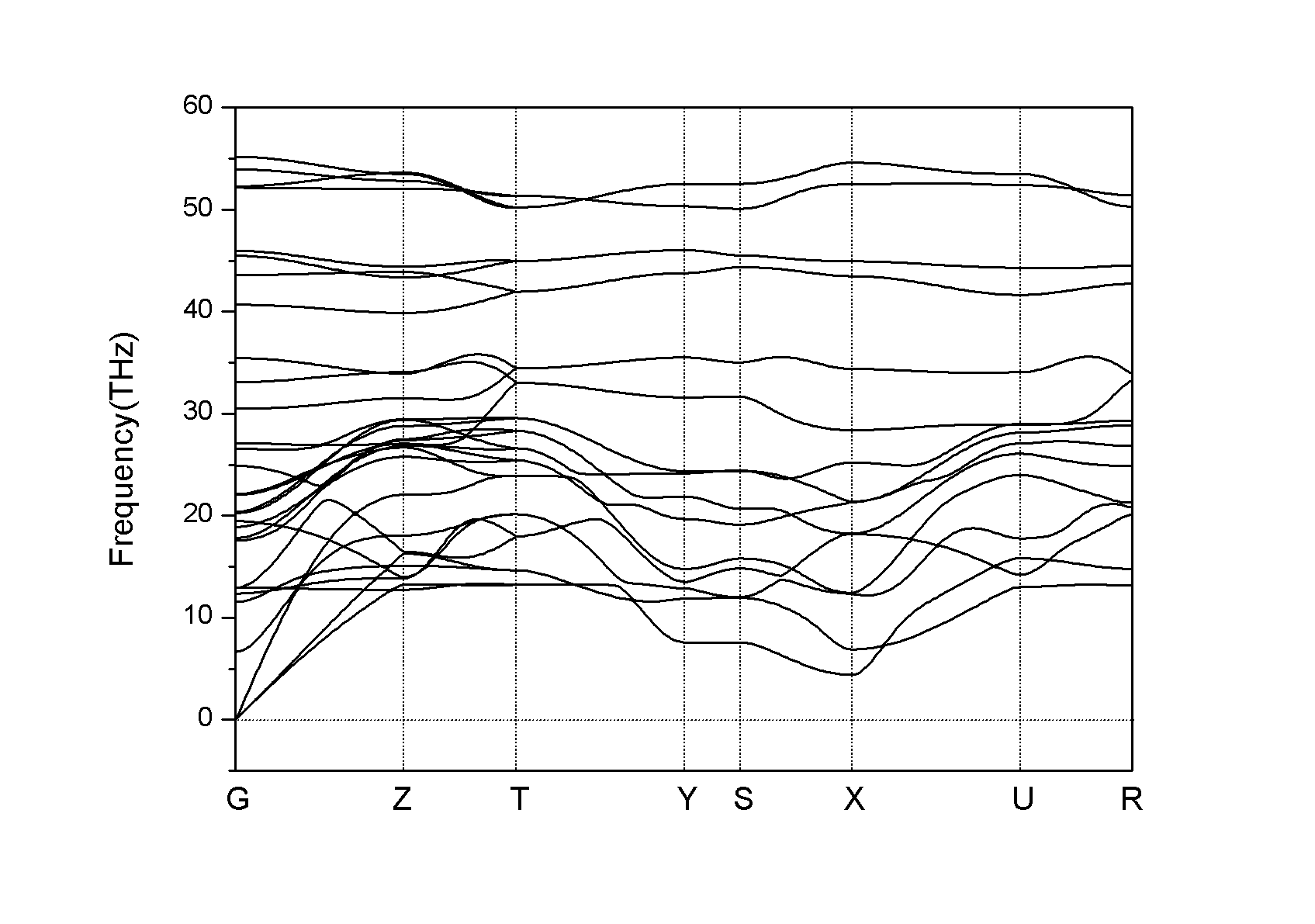


**Figure S10**. Phonon dispersion curves of *Cmmm* phase for FeN_4_ at 300 GPa.


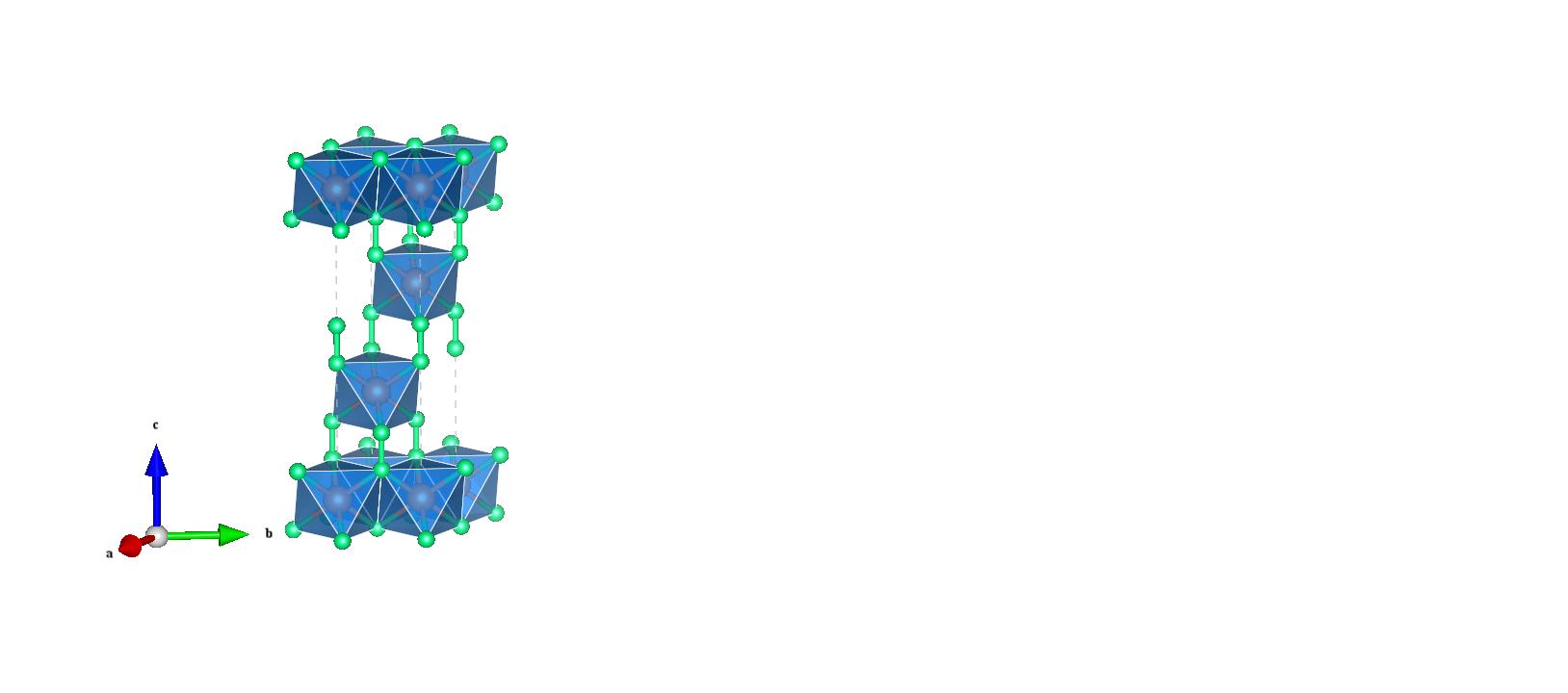


**Figure S11**. Crystal structures of the orthorhombic *R-3m* structure in FeN_2_


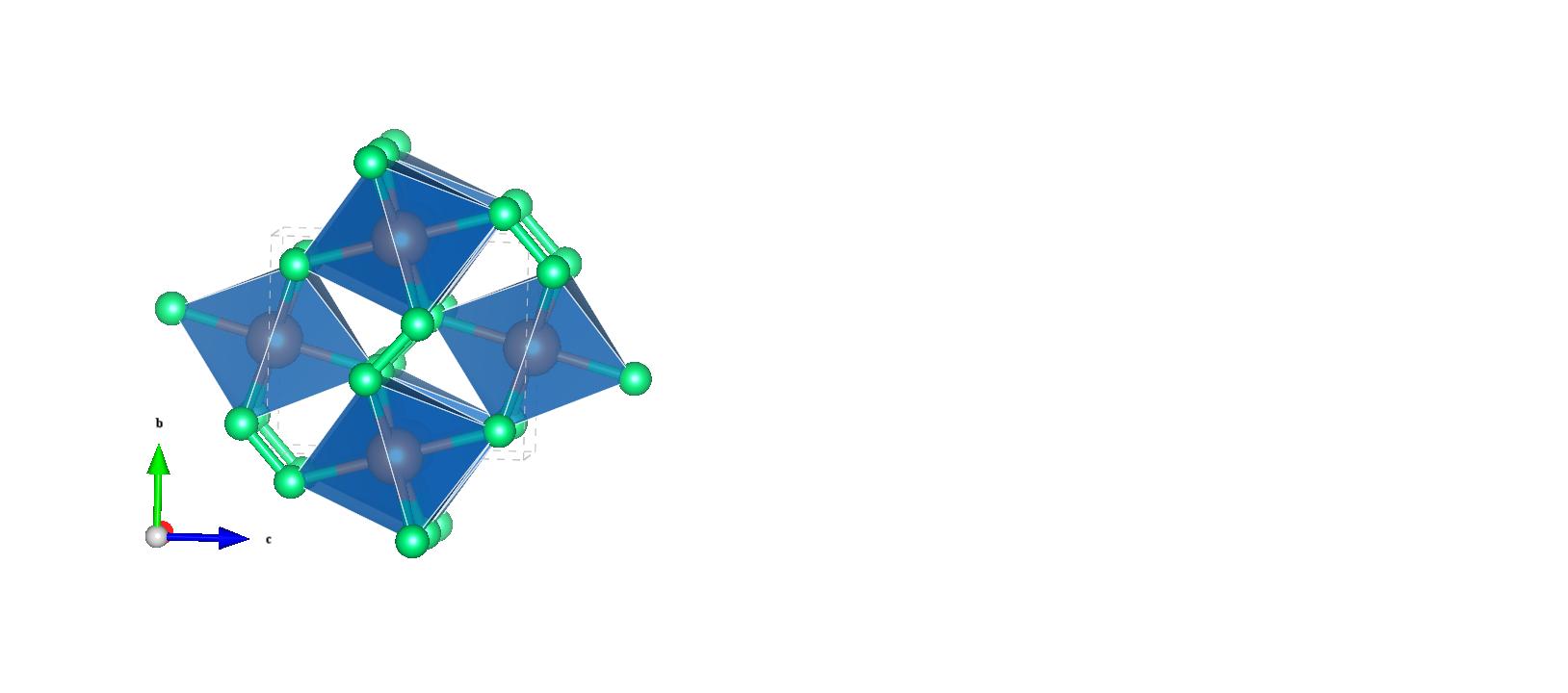


**Figure S12**. Crystal structures of the orthorhombic *Pnnm* structure in FeN_2_


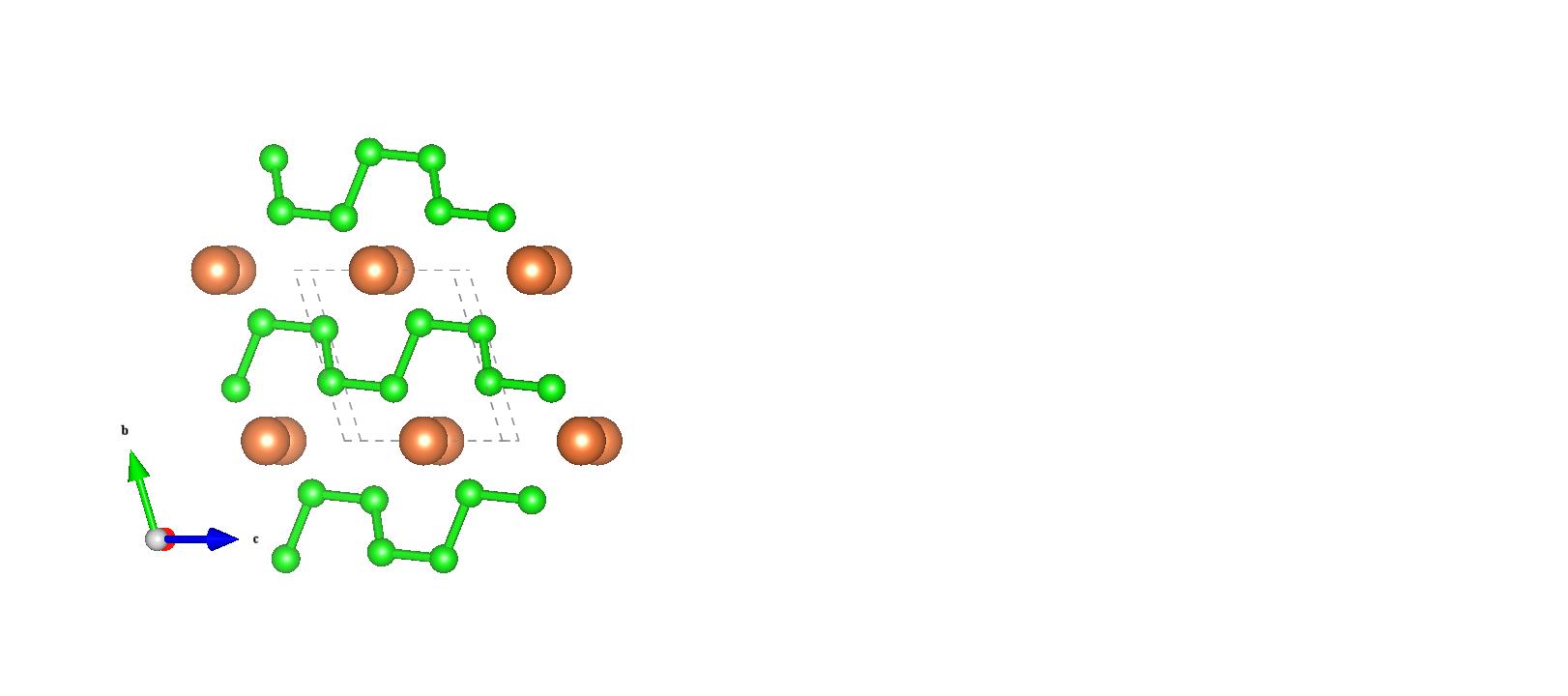

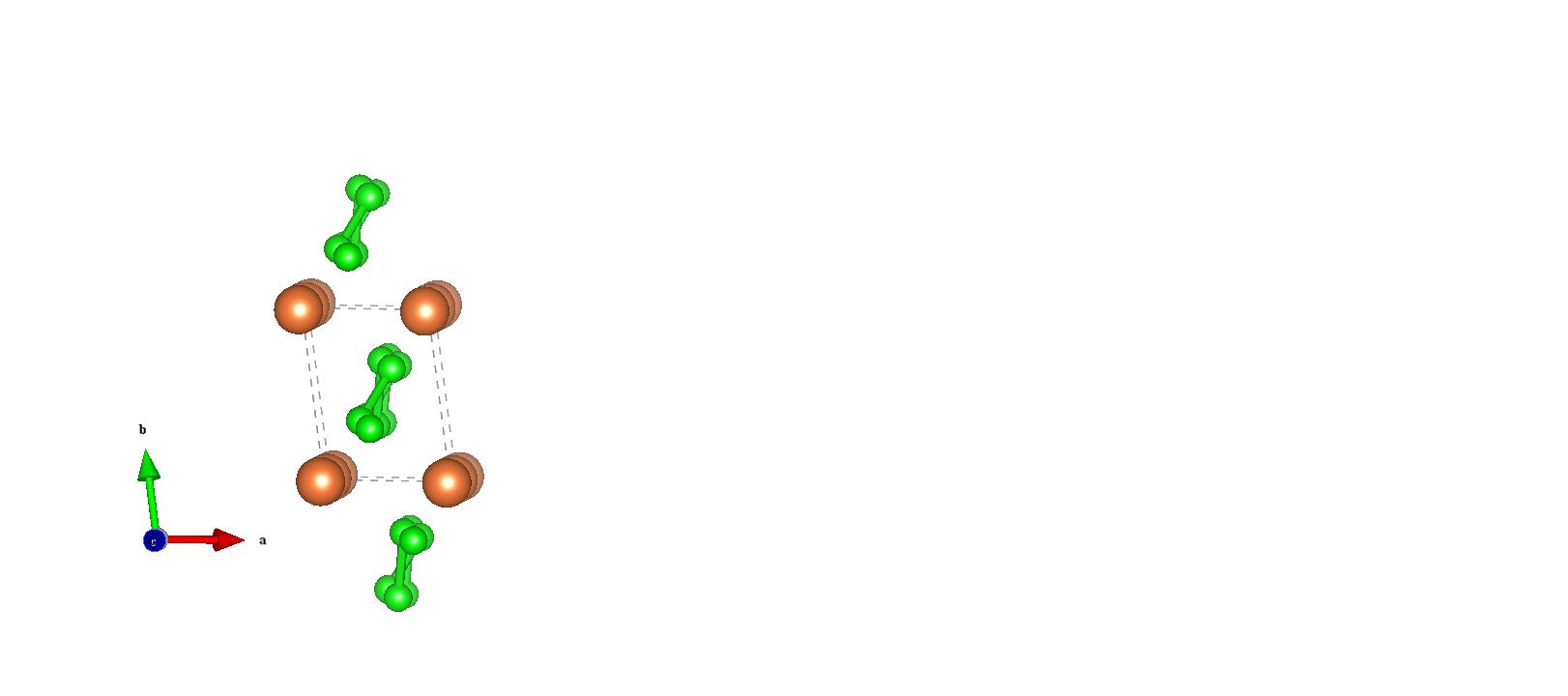

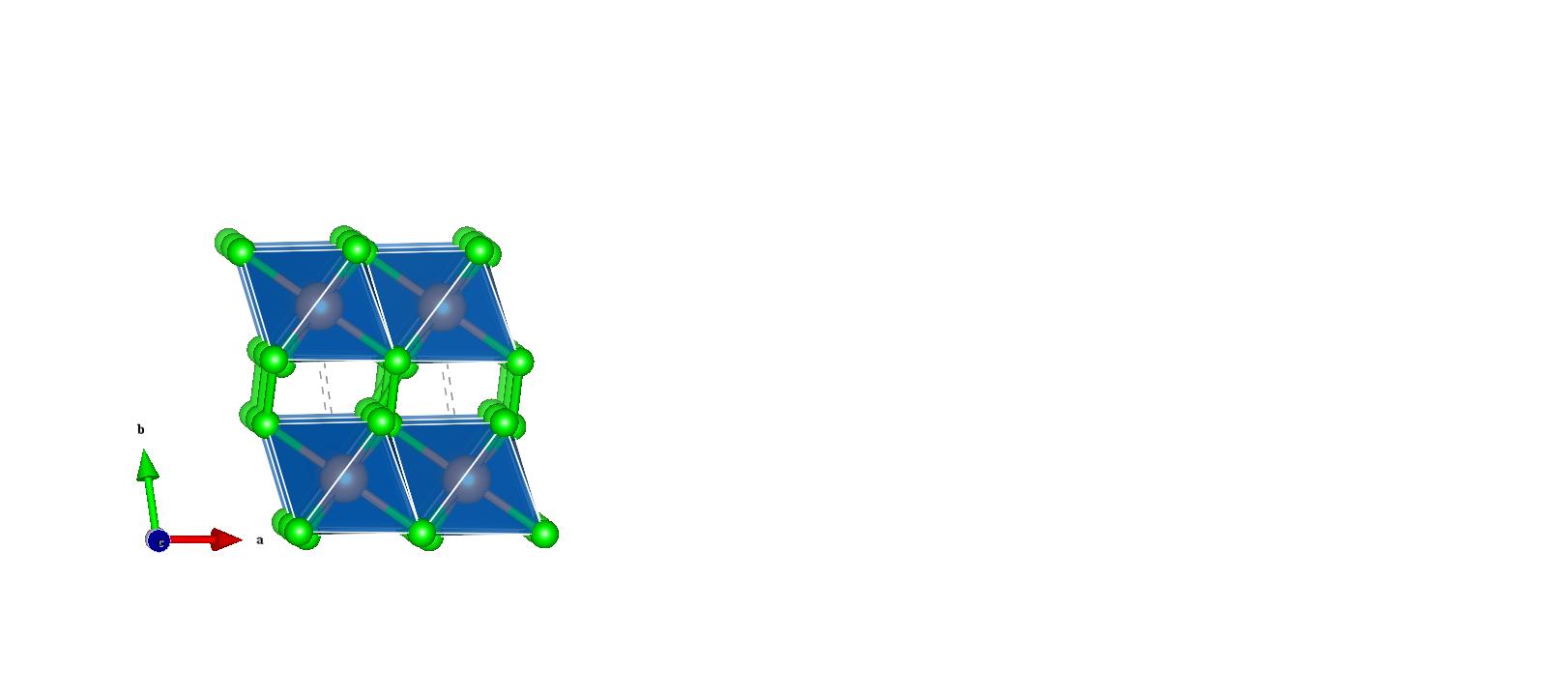


**Figure S13**. The cystal structure of *P*-1 phase of FeN_4_ in the different view.


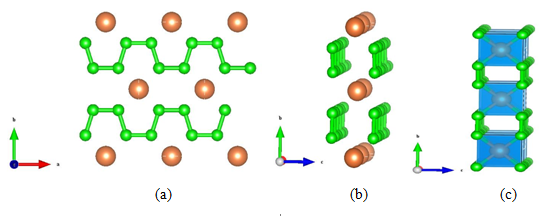


**Figure S14**. The cystal structure of *Cmmm* phase of FeN_4_ in the different view.

**
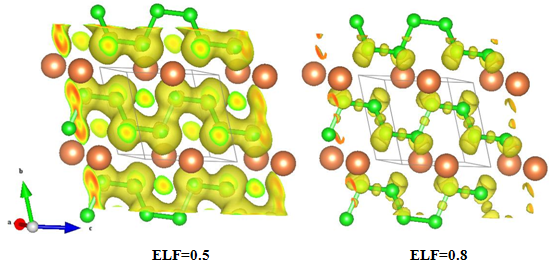
**

**Figure S15**. Calculated ELF of *P*-1 phase for FeN_4_ at 100 GPa.

**
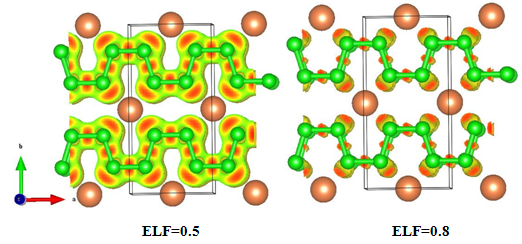
**

**Figure S16**. Calculated ELF of *Cmmm* phase for FeN_4_ at 300 GPa.


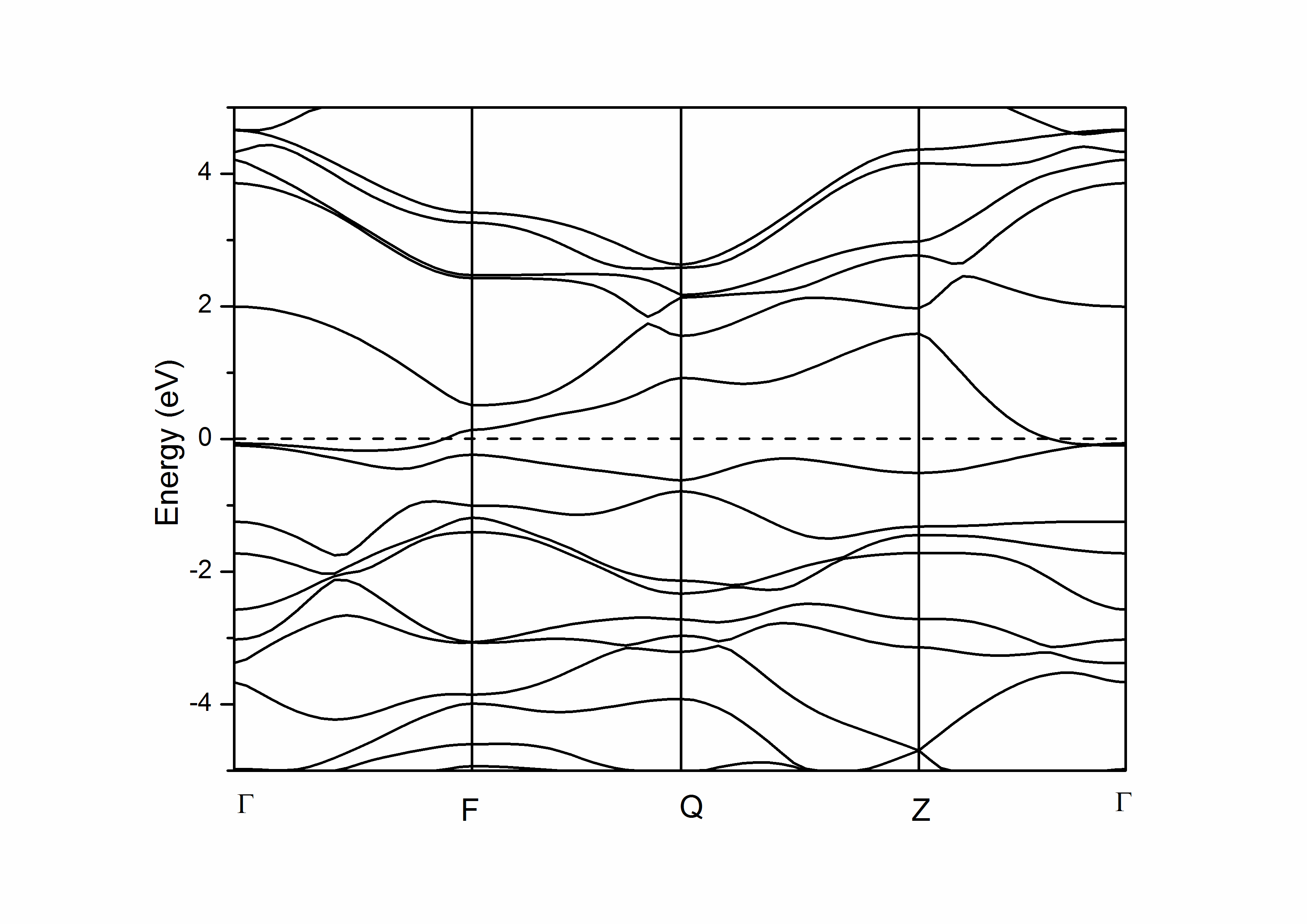


**Figure S17**. Electronic band structure of *P*-1 phase of FeN_4_ at 100 GPa.


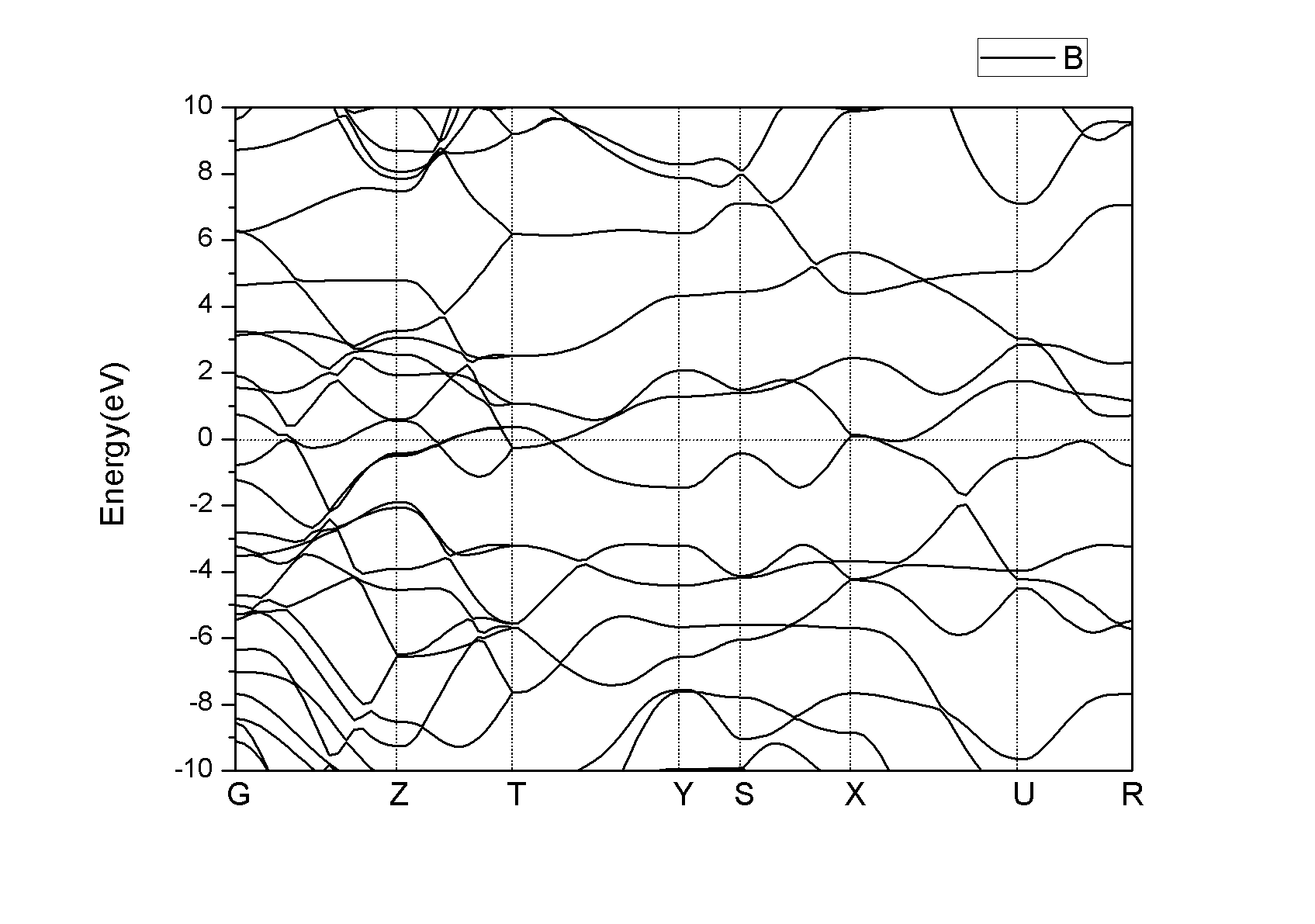


**Figure S18**. Electronic band structure of *Cmmm* phase of FeN_4_ at 300 GPa.

**Table S7**. Character for the point group D_3h_.

| 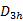=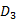⊗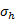 | 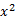+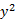,*z*  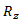 | 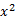+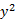,*z*  *z* | xy, yz  R*_x_*, R*_y_* |
| --- | --- | --- | --- |
| *E* | 1 | 1 | 2 |
| 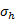 | -1 | -1 | -2 |
| 2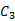 | 1 | 1 | -1 |
| 2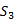 | -1 | -1 | 1 |
| 3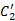 | 1 | -1 | 0 |
| 3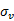 | -1 | 1 | 0 |
|  | 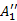 | 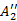 | 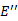 |

**Table S8**. The transformation properties of *p_z_* orbitals locating at N_p_, and N_m_, and Fe1, Fe2 in the point group D_3h_, respectively.

| 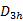=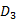⊗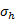 | 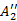⊗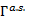for N_p_ | 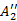⊗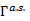for N_m_ | 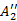⊗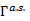for Fe_1_ Fe_2_ |
| --- | --- | --- | --- |
| *E* | 3 | 1 | 2 |
| 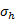 | -3 | -1 | -2 |
| 2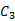 | 0 | 1 | 2 |
| 2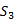 | 0 | -1 | -2 |
| 3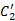 | -1 | -1 | 0 |
| 3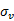 | 1 | 1 | 0 |
|  | =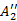+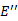 | =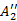 | =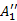+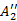 |
